# Surgical Treatment Could Improve Long‐Term Functional Outcomes in Intracerebral Hemorrhage Patients With NIHSS ≥ 10

**DOI:** 10.1111/cns.70458

**Published:** 2025-06-08

**Authors:** Guangshuo Li, Kaijiang Kang, Yi Ju, Yang Du, Anxin Wang, Xiaoli Zhang, Yunyun Xiong, Xingquan Zhao, Wenjuan Wang

**Affiliations:** ^1^ Department of Neurology, Beijing Tiantan Hospital Capital Medical University Beijing China; ^2^ China National Clinical Research Center for Neurological Diseases Beijing China; ^3^ Department of Epidemiology, Beijing Neurosurgical Institute, Beijing Tiantan Hospital Capital Medical University Beijing China; ^4^ Department of Clinical Epidemiology and Clinical Trial Capital Medical University Beijing China; ^5^ Chinese Institute for Brain Research Beijing China

**Keywords:** intracerebral hemorrhage, NIHSS score, outcomes, surgery

## Abstract

**Background and Purpose:**

Surgical treatment has been shown to decrease mortality in patients with intracerebral hemorrhage (ICH) but has not consistently improved functional outcomes. This study aimed to investigate whether surgical treatment could enhance functional outcomes in ICH patients with a National Institutes of Health Stroke Scale (NIHSS) score of 10 or higher.

**Methods:**

This multicenter study included patients from a registry cohort in China with supratentorial ICH, NIHSS scores of 10 or higher, and hematoma sizes of 25 mL or more. Patients were divided into surgical and medical treatment groups. The primary outcome was a modified Rankin Scale (mRS) score of 0–3 at 1 year.

**Results:**

A total of 429 patients were analyzed. The median NIHSS score at admission was 22 [15–29], and the ICH volume was 50 [35.28–72.9] ml. The surgical group had a higher proportion of mRS scores of 0–3 at 1 year compared to the medical group (49.68% vs. 38.32%, *p* = 0.022). Both craniotomy and minimally invasive surgery improved functional outcomes at 1 year (mRS 0–3, craniotomy: OR 2.324, 95% CI 1.266–4.267, *p* = 0.007; minimally invasive surgery: OR 4.884, 95% CI 1.914–12.445, *p* = 0.001).

**Conclusion:**

For patients with supratentorial ICH, NIHSS scores of 10 or higher, and hematoma sizes of 25 mL or more, surgical treatment improved functional outcomes at 1 year compared to medical treatment. The specific subgroup of ICH patients that could benefit from surgical intervention remains controversial.

## Graphical abstract

Surgical treatment can decrease disability (mRS 0–3) in supratentorial ICH patients with an NIHSS score ≥ 10. In the surgical treatment group, supratentorial ICH patients had a more favorable mRS distribution of functional outcomes compared to the medical treatment group. Deep ICH patients are more likely to benefit from surgical treatment compared to those with lobar ICH. Both craniotomy and minimally invasive surgery can improve functional outcomes in ICH.

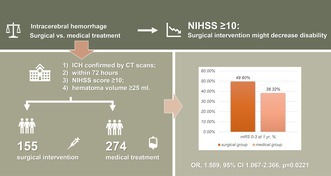

## 1 Introduction

Spontaneous intracerebral hemorrhage (ICH) comprises only 10%–15% of strokes but has a higher mortality rate and greater disability compared to other stroke subtypes [1]. Surgical interventions are recommended as life‐saving therapies for ICH [2]. However, there is still insufficient evidence regarding the efficacy of surgical interventions in reducing disability in ICH patients [3, 4].

In 2022, the American Heart Association (AHA) updated its guidelines on ICH, recommending that patients with supratentorial hematomas larger than 20–30 mL should be considered for surgical interventions [2]. However, clinical trials have not demonstrated the efficacy of surgical interventions in improving functional outcomes compared to conventional medical treatment [3, 4]. Thus, hematoma volume alone may not be the optimal criterion for selecting candidates for surgical interventions. Recently, the NIHSS score has emerged as a crucial tool for predicting functional outcomes in ICH patients [5]. An NIHSS score of 10 or higher has been shown to be a strong predictor of dependence in ICH patients [5] and is also associated with neurological deterioration [6]. Therefore, ICH patients with an NIHSS score of 10 or higher might be at high risk for poor clinical outcomes and may benefit from more aggressive therapy, such as surgical interventions.

This study aimed to investigate whether surgical therapies, compared to conservative medical therapy, could improve functional outcomes in supratentorial ICH patients with an NIHSS score of 10 or higher.

## 2 Method

Our study data was derived from a prospective, multi‐center registry based on 13 stroke centers across China, spanning from January 2014 to September 2016. The study protocol was approved by the Institutional Review Board of Beijing Tiantan Hospital prior to enrollment (IRB no. KY2014‐023‐02). Written informed consent was obtained from the patients or their representatives.

The inclusion criteria for our study were as follows: (1) diagnosis of primary ICH confirmed by CT scans; (2) age ≥ 18; (3) arrival at the hospital within 72 h of symptom onset; (4) admission NIHSS score ≥ 10; and (5) baseline hematoma volume ≥ 25 mL. Patients were excluded if they had (1) infratentorial intracerebral hemorrhage; (2) incomplete follow‐up data at 1 year; (3) ICH due to clear secondary ICH etiology.

### 2.1 Treatment Information

All patients were categorized into two groups: the surgical intervention group and the medical treatment group. Treatment decisions were guided by clinical protocols, including hematoma size, location, and patient comorbidities, as well as shared decision‐making between clinicians and patients. Those who underwent surgery for intracerebral hemorrhage (ICH) during hospitalization were placed in the surgical intervention group. The decision to proceed with surgery, whether craniotomy (hematoma evacuation) or minimally invasive procedures, was made by local surgeons based on standardized criteria. To ensure consistency across different centers, all neurosurgeons involved in the study received uniform training on surgical indications and techniques. Upon completion of this training, they were certified to minimize operator bias. For patients undergoing craniotomy, the procedure began with the patient under general anesthesia. The surgeon made an incision in the scalp, removed a section of the skull (the bone flap), and accessed the brain. The hematoma was carefully located and removed using suction or irrigation techniques. After evacuating the clot and controlling the bleeding, the bone flap was replaced, secured with plates and screws, and the scalp incision was closed. In cases of minimally invasive surgery, a puncture site was identified on the scalp based on the cranial CT scan. Under general anesthesia, a small hole was drilled into the skull using an electric drill at the designated point. A puncture needle and drainage tube were then inserted into the hematoma. The operators aimed to aspirate 30%–50% of the hematoma volume, followed by the administration of urokinase to dissolve and drain the clot over approximately 72 h. Patients who did not undergo any surgical procedures were classified into the medical treatment group.

### 2.2 Clinical and Imaging Data Collection

Trained doctors at local centers collected clinical data, including demographics (age and sex). The pre‐mRS score was used to assess the independent status prior to the index ICH event. Neurological deficit severity was assessed using both the NIHSS score [7] and the GCS score [8]. The ICH score was also recorded. Comorbidities included hypertension, diabetes mellitus, dyslipidemia, atrial fibrillation, ischemic stroke, and ICH, as well as smoking status. Medication history included the use of antiplatelet, anticoagulant, or antihypertensive drugs. Blood sample test results at admission, including INR levels, were documented.

Local radiologists (blinded to clinical information and outcomes) evaluated the radiological data. Hematoma volume was calculated using the ABC/2 formula as previously reported [9]. Hematoma regions were classified as lobar, thalamic, or basal ganglia.

### 2.3 Outcome Assessment

All patients were assessed for functional independence at 1 year after the index ICH. The mRS Score was used to evaluate the functional status of patients by study clinicians who were blinded to the treatment group [10]. The primary outcome of our study was functional independence (mRS 0–3) at 1 year.

### 2.4 Statistical Analyses

Continuous variables were expressed as mean ± SD for normally distributed data and median [interquartile range] for non‐normally distributed data. Categorical variables were expressed as numbers (percentages). In univariate analyses, normally distributed data were compared using the Student's *t*‐test, and non‐normally distributed data were compared using non‐parametric tests. Categorical variables were compared using the *χ*2 test or Fisher's exact test, as appropriate. Patients were dichotomized into the surgical intervention group and the conservative medical group to compare disability status and other outcomes. To address potential selection bias and potential covariates, different multivariable analyses were performed, reporting odds ratios (ORs), 95% confidence intervals (CIs), and *p*‐values, to balance baseline characteristics between the surgical and medical treatment groups adjusting for age, sex, hematoma size, GCS Score, NIHSS Score, and covariates with *p* < 0.05. A shift analysis was used to compare the mRS distribution at 1 year between the surgical intervention and medical groups. A survival curve was displayed to show the cumulative death risk at 1 year. Subgroup analyses were performed to test the treatment effect across different patient groups. All statistical analyses were performed using SAS software (version 9.4; SAS Institute Inc.), with statistical significance defined as a two‐sided *p* < 0.05.

## 3 Results

**FIGURE 1:**
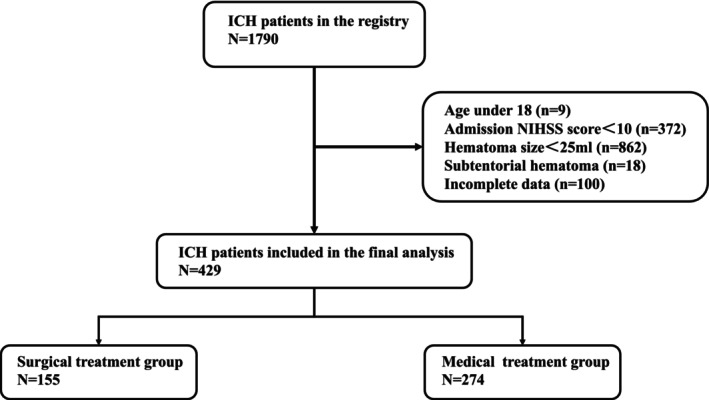
Flow‐chart. ICH, intracerebral hemorrhage; NIHSS, National Institutes of Health Stroke Scale.

A total of 429 ICH patients were included in our final analyses, with 155 patients receiving surgical interventions (Figure 1). The mean age was 58.49 ± 13.77 years, and 314 (73.19%) patients were male. The median NIHSS Score at admission was 22 [15–29], and the median GCS Score was 9 [5–13]. The ICH volume was 50 [35.28–72.9] mL. Among the included patients, 38.86% (157 patients) had lobar hemorrhage, and 90.82% (386 patients) were classified as having ICH due to hypertension. The mortality rate at 1 year was 41.26%, and the percentage of patients achieving independence (mRS 0–3) was 42.42% (Table 1).

**TABLE 1:** Demographic and clinical characteristics between surgical interventions vs. medical treatment alone.

	Overall (*n* = 429)	Surgical treatment group (*n* = 155)	Medical treatment group (*n* = 274)	*p*
Age, years (mean (SD))	58.49 ± 13.77	56.56 ± 12.58	59.58 ± 14.30	0.06
Male, *n* (%)	314 (73.19)	119 (76.77)	195 (71.17)	0.208
Pre‐mRS, *n* (%)				0.62
0	358 (83.45)	125 (80.65)	233 (85.04)	
1	36 (8.39)	17 (10.97)	19 (6.93)	
2	17 (3.96)	5 (3.23)	12 (4.38)	
3	3 (0.70)	1 (0.65)	2 (0.73)	
4	12 (2.8)	6 (3.87)	6 (2.19)	
5	3 (0.70)	1 (0.65)	2 (0.73)	
Time interval between onset and admission, hours, median [interquartile range]	3.39[1.77–8.25]	2.92[1.50–6.60]	3.92[1.98–9.58]	0.007
NIHSS score at admission, median [interquartile range]	22 [15–29]	20.5 [16–26]	23 [15–30]	0.282
GCS at admission, median [interquartile range]	9 [5–13]	8 [5–12]	9.5 [5–13]	0.23
ICH‐score, median [interquartile range]	2 [0–4]	2 [1–2]	2 [1–3]	0.783
Hypertension, *n* (%)	317 (74.24)	123 (79.35)	194 (71.32)	0.068
Diabetes mellitus, *n* (%)	66 (15.38)	25 (16.13)	41 (14.96)	0.748
Dyslipidemia, *n* (%)	36 (8.39)	8 (5.16)	28 (10.22)	0.07
Atrial fibrillation, *n* (%)	7 (1.63)	1 (0.65)	6 (2.19)	0.19
Ischemic stroke, *n* (%)	64 (14.92)	19 (12.26)	45 (16.42)	0.24
Intracerebral hemorrhage, *n* (%)	14 (3.26)	6 (3.87)	8 (2.92)	0.59
Smoking, *n* (%)				0.76
Never	193 (44.99)	73 (47.10)	120 (43.80)	
Current	156 (36.36)	56 (36.13)	100 (36.50)	
Cessation	48 (11.19)	17 (10.97)	31 (11.31)	
Unknown	32 (7.46)	9 (5.81)	23 (8.39)	
Alcohol abuse, *n* (%)	171 (39.86)	64 (41.29)	107 (39.05)	0.88
Antiplatelet agents, *n* (%)	64 (14.92)	26 (16.77)	38 (13.87)	0.007
Anticoagulant agents, *n* (%)	4 (0.93)	1 (0.23)	3 (1.09)	0.03
Antihypertensive agents, *n* (%)	144 (33.57)	56 (36.13)	88 (32.12)	0.286
Antidiabetic agents, *n* (%)	33 (7.69)	11 (7.10)	22 (8.03)	0.102
Systolic pressure at admission, median [interquartile range], mmHg	175 [157–195]	175 [160–200]	173.5 [152–192.5]	0.102
INR, median [interquartile range]	0.97 [0.92–1.03]	0.98 [0.93–1.03]	0.96 [0.91–1.03]	0.073
Admission hematoma volume [interquartile range], mL	50 [35.28–72.9]	56.64 [41.8–80]	48 [31.8–71.3]	0.025
Intraventricular hemorrhage^a^, *n* (%)	192 (53.04)	90 (60.81)	102 (47.66)	0.014
Hematoma region^b^, *n* (%)				0.01
Lobar	157 (38.86)	46 (30.07)	111 (44.22)	
Basal ganglia	236 (58.42)	104 (67.97)	132 (52.59)	
Thalamus	11 (2.72)	3 (1.96)	2 (3.19)	
Subtype of primary intracerebral hemorrhage^c^, *n* (%)				0.398
Hypertensive	386 (90.82)	142 (92.81)	244 (89.71)	
Cerebral Amyloid Angiopathy	15 (3.53)	3 (1.96)	12 (4.41)	
Other/unknown	24 (5.65)	8 (5.23)	16 (5.88)	
In‐hospital mortality, *n* (%)	108 (25.17)	18 (11.61)	90 (32.85)	0.001
Hospitalization length, median [interquartile range], days	20 [12–27]	22.5 [14–29]	18 [8–25]	0.002
30‐d death, *n* (%)	149 (34.73)	28 (18.06)	121 (44.16)	0.001
30d mRS [interquartile range]	5 [4–6]	4 [4–5]	5 [4–6]	0.001
30d mRS 0–3, *n* (%)	82 (19.11)	26 (16.77)	56 (20.44)	0.354
90‐d death, *n* (%)	158 (36.83)	34 (21.94)	124 (45.26)	0.001
90d mRS [interquartile range]	4 [3–6]	4 [3–5]	5 [3–6]	0.006
90d mRS 0–3, *n* (%)	130 (30.37)	46 (29.87)	84 (30.66)	0.865
Death at 1 year, *n* (%)	177 (41.26)	44 (28.39)	133 (48.54)	0.001
mRS at 1 year [interquartile range]	4 [3–6]	4 [3–6]	5 [3–6]	0.032
mRS 0–3 at 1 year, *n* (%)	182 (42.42)	77 (49.68)	105 (38.32)	0.022

Abbreviations: GCS, glascow coma scale; INR, international normalized ratio; mRS, modified Rankin Scale; NIHSS, national institutes of health stroke scale.

^a^ Missing 67 patients.

^b^ Missing 25 patients.

^c^ Missing 4 patients.

**FIGURE 2:**
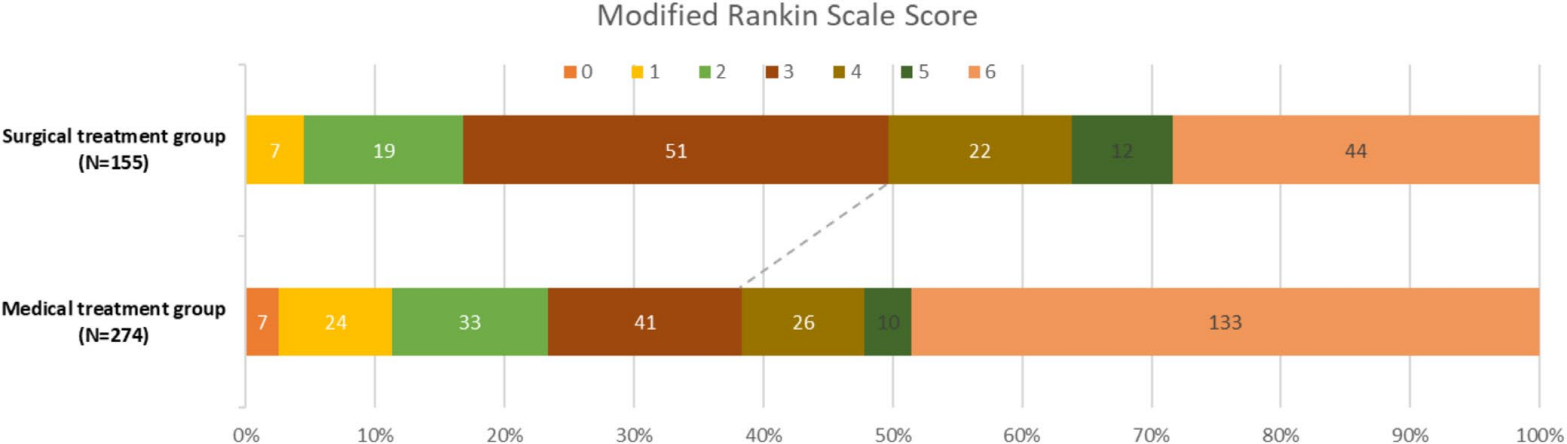
mRS distribution between surgical and medical group.

**FIGURE 3:**
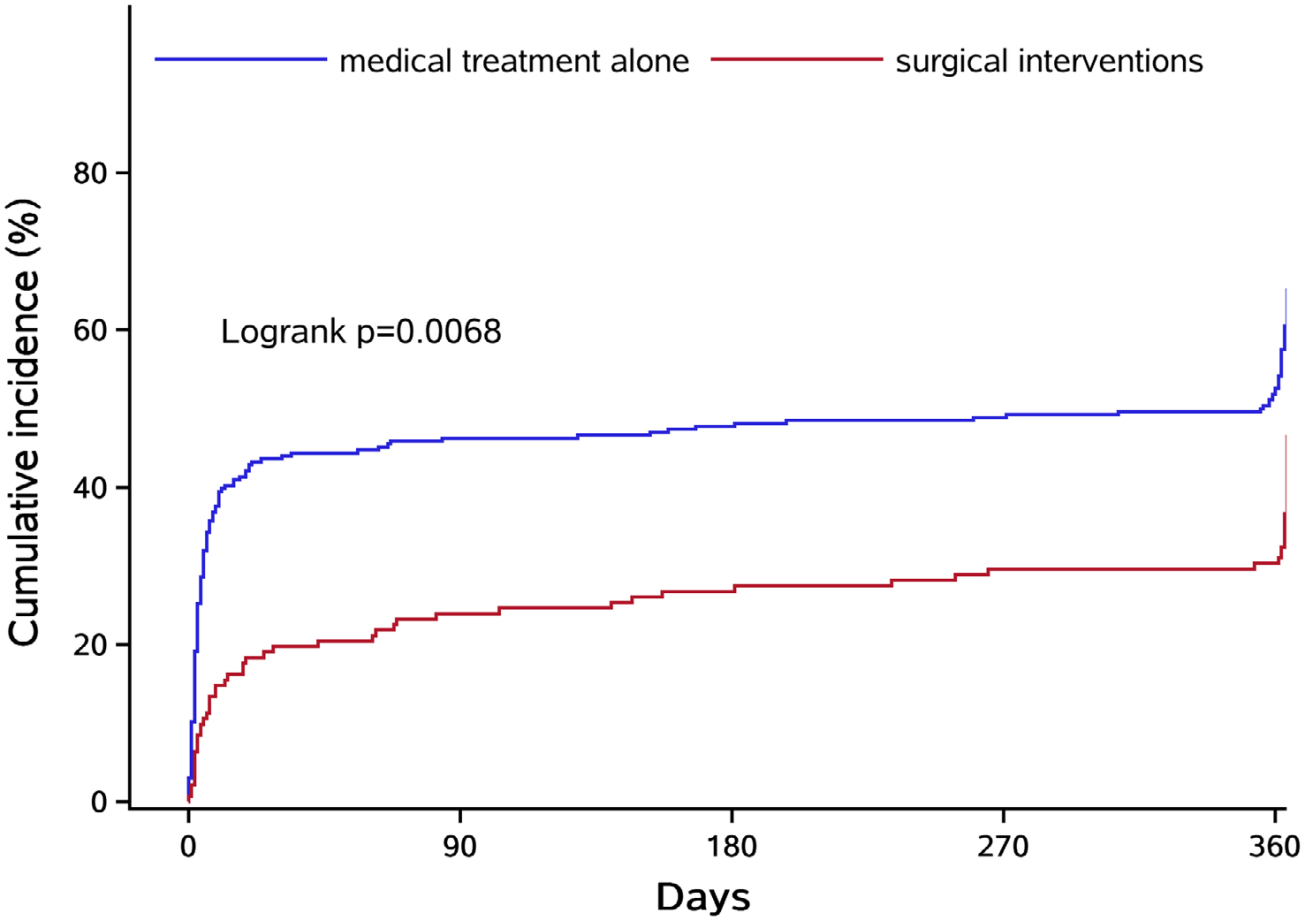
Survival curve.

Compared with the standard medical group, the surgical intervention group had shorter time intervals from onset to admission (2.92 [1.50–6.60] vs. 3.92 [1.98–9.58] hours, *p* = 0.007). In the surgical intervention group, a higher percentage of patients were on antiplatelet drugs (16.77% vs. 13.87%, *p* = 0.03). The baseline hematoma volume was higher in the surgical group compared to the standard medical group (56.64 [41.8–80] vs. 48 [31.8–71.3] mL, *p* = 0.003). More patients survived to discharge if they received surgical interventions (88.39% vs. 67.15%, *p* < 0.001). The surgical intervention group also had a longer length of hospital stay compared to the standard medical group (22.5 [14–29] vs. 18 [8–25] days, *p* < 0.001). At 1 year, the surgical intervention group had a lower mortality rate compared to the standard medical group (28.39% vs. 48.54%, *p* < 0.001), which was also shown in a survival curve (Figure 2). Additionally, the surgical intervention group had better functional outcomes at 1 year, including a higher independence rate (49.68% vs. 38.32%, *p* = 0.022) and a lower median mRS Score (4 [3–6] vs. 5 [3–6], *p* = 0.032) (Table 1 and Figure 3).

Several multiple logistic models were established to eliminate the potential effects of covariates on functional outcomes. After adjusting for different factors, surgical intervention remained independently associated with functional independence at 1 year (crude OR, 1.589, 95% CI 1.067–2.366, *p* = 0.0221; model 2: adjusted OR, 1.46, 95% CI 0.952–2.239, *p* = 0.083; model 3: adjusted OR, 2.765, 95% CI 1.594–4.797, *p* = 0.001; model 4: adjusted OR, 2.08, 95% CI 1.231–3.514, *p* = 0.006). (Table 2; Table S1) Besides, sensitivity analyses were performed after excluding patients with a mRS score 4–5 before the index ICH, considering that functional independence (mRS 0–3) at 1 year. Surgical interventions remained significantly beneficial in improving functional independence, compared with medical treatment (Table S2).

**TABLE 2:** Multivariable logistic regression analyses.

Outcome	Model	OR	95% CI	*p*
90d mRS 0–3	Model 1	0.963	0.627–1.481	0.865
	Model 2	0.852	0.544–1.334	0.484
	Model 3	1.289	0.761–2.184	0.346
	Model 4	1.33	0.769–2.301	0.307
1 year mRS 0–3	Model 1	1.589	1.067–2.366	0.023
	Model 2	1.46	0.952–2.239	0.083
	Model 3	2.765	1.594–4.797	0.001
	Model 4	2.08	1.231–3.514	0.006
1 year mortality	Model 1	0.776	0.633–0.952	0.015
	Model 2	0.773	0.629–0.951	0.015
	Model 3	0.536	0.425–0.677	0.001
	Model 4	0.322	0.485–0.799	0.001

*Note:* Model 2: adjusted for age and sex. Model 3: adjusted for age, sex, hematoma size and GCS score. Model 4: adjusted for age, sex and covariates with *p* < 0.05 in Table 1.

**FIGURE 4:**
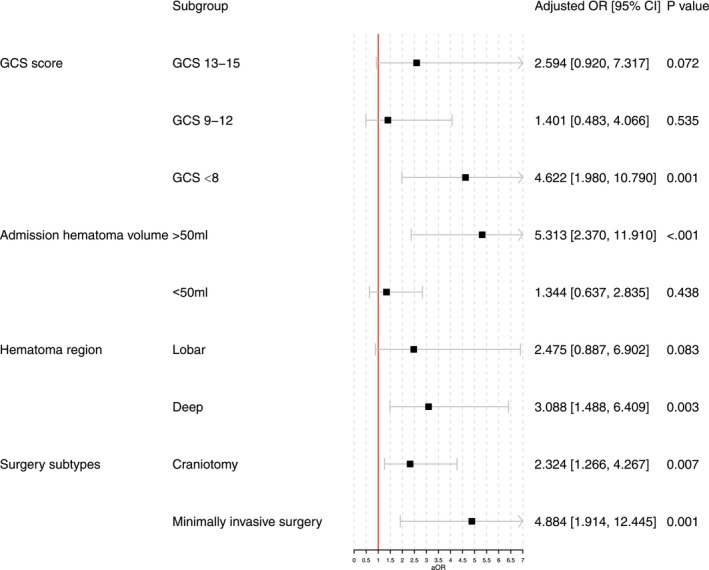
Subgroup analyses. OR, odds ratio; CI, confidence intervals; GCS, Glasgow coma scale.

Subgroup analyses were further conducted to investigate whether the benefit from surgical intervention differed across various subgroups, adjusting for age, sex, hematoma size, and admission NIHSS score. Patients with GCS < 8, baseline hematoma < 50 mL, or deep hematomas were more likely to benefit from surgical treatment in terms of improving functional independence (mRS 0–3) at 1 year. Both craniotomy and minimally invasive surgery were found to improve functional independence (Figure 4).

## 4 Discussion

In this study, ICH patients with an NIHSS score ≥ 10 showed a more favorable functional outcome in the surgical group compared to those in the conservative medical group. A higher proportion of mRS 0–3 was observed in the surgical group at 1 year, and this association remained significant even after adjusting for covariates. The surgical group also exhibited a higher median mRS score at 1 year. Additionally, the 1‐year mortality rate was lower in the surgical group. Patients with large hematomas and severe neurological impairments have a higher risk of neurological deterioration and poor prognosis, necessitating more aggressive therapies, including surgical interventions [11]. Our study found that the NIHSS score could be a valid tool to screen for ICH patients who would benefit from surgical therapy by improving functional outcomes, a topic rarely investigated before.

Our study showed that surgical interventions were favored in ICH patients with NIHSS ≥ 10 because of a favorable functional outcome at 1 year. The MISTIE III trial was the largest clinical trial to investigate the efficacy and safety of minimally invasive surgery in supratentorial ICH, with a majority of deep hematomas in the included population similar to our study [4]. At 1 year, 44% of the patients in the surgical group and 42% of the patients in the medical group achieved mRS 0–3. In our study, the proportion of mRS 0–3 was 38.32% in the medical group, likely due to higher baseline NIHSS scores and larger hematoma sizes compared to the MISTIE III trial. However, the surgical group showed a higher proportion of mRS 0–3 than the MISTIE III trial. Considering the higher NIHSS scores and larger hematoma sizes in our study, a potential explanation might be a higher proportion of deep hematomas in our study compared to the MISTIE III, where hematoma location favored surgical therapy more than lobar hemorrhage. We defined mRS 0–3 at 1 year rather than 90 days as the main study outcome. Given the more severe neurological deficits in ICH compared to ischemic stroke, longer rehabilitation and neurological recovery times might be necessary [12, 13].

Our study found that mortality was significantly decreased in the surgical group compared to the medical group. This decreased mortality due to surgical treatment was also shown in the MISTIE III [4] and STICH II [3] trials, though some single‐center cohorts did not [14]. The surgical group in our study had a slightly higher mortality at 1 year (28.39%) compared to the MISTIE III trial [4], which could be due to higher NIHSS scores and larger hematoma sizes in our study. As recommended by AHA/ASA in 2022 [2], surgical interventions can be used as a life‐saving therapy in clinical practice.

Our study aimed to determine whether the NIHSS score could be used to identify ICH candidates who might benefit from surgical interventions. Our findings suggest that ICH patients with an NIHSS score of ≥ 10 may experience reduced long‐term disability following surgical treatment. Unlike previous clinical trials, which primarily focused on either craniotomy or minimally invasive surgery, our study evaluated the efficacy of both procedures. Three major clinical trials—MISTIE III [4], STICH II [3], and ENRICH [15]—have explored the safety and efficacy of surgical interventions in ICH patients compared to medical treatment. However, our study differed in several key aspects. For instance, MISTIE III [4] exclusively evaluated minimally invasive surgery, while STICH II [3] only included craniotomy. Moreover, STICH II [3] was limited to patients with lobar hematomas, whereas our study included both lobar and deep hematomas. The ENRICH [15] trial was the only one among the three that demonstrated the effectiveness of minimally invasive surgery in reducing disability. However, ENRICH [15] used the utility‐weighted modified Rankin Scale (mRS) as its primary efficacy measure, while our study employed the more widely used mRS 0–3. Additionally, our study had a longer follow‐up period for assessing disability outcomes (365 days vs. 180 days in ENRICH [15]). Notably, while ENRICH [15] showed greater efficacy in lobar hematomas, our study demonstrated significant benefits in deep hematomas with both surgical approaches. In summary, while the three trials [3, 4, 15] provided high‐level evidence, our registry study offers unique insights due to differences in patient inclusion criteria, types of surgical interventions, and outcome assessments.

Though the GCS score was previously a more frequent tool for evaluating the severity of ICH, the NIHSS score showed superior predictive value for clinical outcomes in ICH, independent of hematoma size [5]. Even compared to other ICH‐related scales, the NIHSS score showed non‐inferiority in predicting different clinical outcomes. A potential explanation for the superiority of the NIHSS score over the GCS score is its more extensive coverage of a wider range of neurological functions. The NIHSS score evaluates not only consciousness level but also upper‐ and lower‐limb motor functions (with a wider score range), which are highly related to future independent status. By contrast, the GCS score merely emphasizes consciousness level and reflex function. Compared to previous combined analyses supporting the key role of NIHSS in ICH [5], our study confirmed its role in ICH patients with larger hematoma sizes and those who received surgical interventions, as well as its correlation with long‐term functional outcomes.

The AHA updated the ICH guidelines in 2022 and recommended assessing ICH patients for the benefits of surgical interventions mainly based on hematoma location and size [2], which also predict neurological deterioration and long‐term prognosis [11]. Recent data showed the necessity of the NIHSS score as a predictor of functional outcomes in ICH [5]. Our study confirmed the new application of the NIHSS score in surgical interventions for ICH. Hematoma size proved to be a key parameter in ICH trials [16]. Further attention has been raised on the NIHSS score in addition to hematoma size in ICH. A potential explanation is the mediation effect of the NIHSS score. A multi‐center cohort confirmed the 14%–30% effect that mediated the association between hematoma size and functional outcomes in ICH [17]. The DAWN trial proposed an NIHSS‐infarction size mismatch model as a sign of ischemic penumbra, known as the therapeutic target, in ischemic stroke [18]. Ischemic penumbra would become core infarction if ischemia persisted but could revert to normal tissue if recanalization occurred [19, 20]. Hence, the NIHSS‐infarction size mismatch suggested unstable ischemic tissue that could be saved and serve as a therapeutic target of recanalization therapy. However, in ICH, surgical interventions for hematoma evacuation should aim at stable hematomas with a low risk of expansion, suggesting an ‘NIHSS‐hematoma size match’, as shown in our study (NIHSS score ≥ 10 and hematoma size ≥ 25 mL).

Several limitations should be acknowledged. Firstly, as a prospective registry study, our conclusions are not as robust as those derived from randomized controlled trials. Treatment selection was not based on a randomized algorithm, raising concerns regarding potential bias. Multivariable logistic regression models, sensitivity, and subgroup analyses were used to adjust for different covariates. Secondly, hematoma size was calculated manually in our study. As the inclusion of our study began in 2014, few automatic software solutions had proven accurate for measuring hematoma size. All radiologists were required to receive training before imaging assessment. Furthermore, our study reflects real‐world clinical practice, offering practical insights that complement the findings of randomized controlled trials. Thirdly, detailed data on surgical interventions were not provided as most doctors collecting the study data were neurologists, not neurosurgeons. Our study was not initiated to demonstrate the efficacy of specific surgical interventions but to introduce an effective tool to select patients who could benefit from surgical intervention (NIHSS score). Future randomized trials with rigorous designs might provide evidence with greater statistical power.

### 4.1 Conclusion

In conclusion, our study demonstrates that surgical interventions significantly improve functional outcomes and reduce mortality at 1 year for ICH patients with an NIHSS score ≥ 10, suggesting that NIHSS can serve as a crucial tool in identifying candidates for surgical treatment. This finding underscores the need for incorporating NIHSS scores alongside hematoma size in clinical decision‐making for ICH management.

## Disclosure

The authors have nothing to report.

## Ethics Statement

Approved by the ethics committee at Beijing Tiantan Hospital and all other participating centers.

## Consent

Written informed consent was obtained from participants or their legally authorized representatives.

## Conflicts of Interest

The authors declare no conflicts of interest.

## Supporting information


**Table S1.** Multivariable logistics models adjusted for NIHSS score.
**Table S2.** Multivariable logistics models excluding patients with a mRS Score 4–5 before the index ICH.

## Data Availability

Data are available from the corresponding author upon reasonable request.
