# Supplementary material for: Surgical Treatment Could Improve Long‐Term Functional Outcomes in Intracerebral Hemorrhage Patients With NIHSS ≥ 10
Source: CNS Neurosci Ther. 2025 Jun 8;31(6):e70458. doi: 10.1111/cns.70458 (PMC12146580; doi:10.1111/cns.70458)
Supplement: Supplementary file 1 — Table S1. Multivariable logistics models adjusted for NIHSS score. Table S2. Multivariable logistics models excluding patients with a mRS Score 4–5 before the index ICH. [file CNS-31-e70458-s001.docx]

Table S1. Multivariable logistics models adjusted for NIHSS score

| Outcome | Model | OR | 95%CI | p |
| --- | --- | --- | --- | --- |
| 1 yrs mRS 0-3 | Model5 | 1.683 | 1.069-2.649 | 0.0246 |
|  | Model6 | 2.052 | 1.268-3.322 | 0.0034 |
|  | Model7 | 1.953 | 1.152-3.311 | 0.0129 |

Model5：adjusted for NIHSS score

Model6：adjusted for NIHSS score，admission hematoma size

Model7：adjusted for NIHSS score，admission hematoma size，age，sex

Table S2. Multivariable logistics models excluding patients with a mRS score 4-5 before the index ICH

| Outcome | Model | OR | 95%CI | p |
| --- | --- | --- | --- | --- |
| 1 yrs mRS 0-3 | Model1 | 1.480 | 0.989-2.214 | 0.0566 |
|  | Model2 | 1.282 | 0.832-1.976 | 0.2603 |
|  | Model3 | 2.518 | 1.438-4.409 | 0.0012 |
|  | Model4 | 2.203 | 1.266-3.834 | 0.0052 |
|  | Model5 | 1.698 | 1.068-2.698 | 0.0251 |
|  | Model6 | 2.079 | 1.270-3.402 | 0.0036 |
|  | Model7 | 1.971 | 1.150-3.377 | 0.0136 |

Model 2: adjusted for age and sex

Model 3: adjusted for age, sex, hematoma size and GCS score

Model 4: adjusted for age, sex and covariates with p<0.05 in table 1

Model5：adjusted for NIHSS score

Model6：adjusted for NIHSS score，admission hematoma size

Model7：adjusted for NIHSS score，admission hematoma size，age，sex
